# Machine learning and deep learning predictive models for type 2 diabetes: a systematic review

**DOI:** 10.1186/s13098-021-00767-9

**Published:** 2021-12-20

**Authors:** Luis Fregoso-Aparicio, Julieta Noguez, Luis Montesinos, José A. García-García

**Affiliations:** 1 School of Engineering and Sciences, Tecnologico de Monterrey, Av Lago de Guadalupe KM 3.5, Margarita Maza de Juarez, 52926 Cd Lopez Mateos, Mexico; 2 School of Engineering and Sciences, Tecnologico de Monterrey, Ave. Eugenio Garza Sada 2501, 64849 Monterrey, Nuevo Leon Mexico; 3 Hospital General de Mexico Dr. Eduardo Liceaga, Dr. Balmis 148, Doctores, Cuauhtemoc, 06720 Mexico City, Mexico

**Keywords:** Diabetes, Machine learning, Deep learning, Review, Electronic health records

## Abstract

Diabetes Mellitus is a severe, chronic disease that occurs when blood glucose levels rise above certain limits. Over the last years, machine and deep learning techniques have been used to predict diabetes and its complications. However, researchers and developers still face two main challenges when building type 2 diabetes predictive models. First, there is considerable heterogeneity in previous studies regarding techniques used, making it challenging to identify the optimal one. Second, there is a lack of transparency about the features used in the models, which reduces their interpretability. This systematic review aimed at providing answers to the above challenges. The review followed the PRISMA methodology primarily, enriched with the one proposed by Keele and Durham Universities. Ninety studies were included, and the type of model, complementary techniques, dataset, and performance parameters reported were extracted. Eighteen different types of models were compared, with tree-based algorithms showing top performances. Deep Neural Networks proved suboptimal, despite their ability to deal with big and dirty data. Balancing data and feature selection techniques proved helpful to increase the model’s efficiency. Models trained on tidy datasets achieved almost perfect models.

## Introduction

Diabetes mellitus is a group of metabolic diseases characterized by hyperglycemia resulting from defects in insulin secretion, insulin action, or both [1]. In particular, type 2 diabetes is associated with insulin resistance (insulin action defect), i.e., where cells respond poorly to insulin, affecting their glucose intake [2]. The diagnostic criteria established by the American Diabetes Association are: (1) a level of glycated hemoglobin (HbA1c) greater or equal to 6.5%; (2) basal fasting blood glucose level greater than 126 mg/dL, and; (3) blood glucose level greater or equal to 200 mg/dL 2 h after an oral glucose tolerance test with 75 g of glucose [1].

Diabetes mellitus is a global public health issue. In 2019, the International Diabetes Federation estimated the number of people living with diabetes worldwide at 463 million and the expected growth at 51% by the year 2045. Moreover, it is estimated that there is one undiagnosed person for each diagnosed person with a diabetes diagnosis [2].

The early diagnosis and treatment of type 2 diabetes are among the most relevant actions to prevent further development and complications like diabetic retinopathy [3]. According to the ADDITION-Europe Simulation Model Study, an early diagnosis reduces the absolute and relative risk of suffering cardiovascular events and mortality [4]. A sensitivity analysis on USA data proved a 25% relative reduction in diabetes-related complication rates for a 2-year earlier diagnosis.

Consequently, many researchers have endeavored to develop predictive models of type 2 diabetes. The first models were based on classic statistical learning techniques, e.g., linear regression. Recently, a wide variety of machine learning techniques has been added to the toolbox. Those techniques allow predicting new cases based on patterns identified in training data from previous cases. For example, Kälsch et al. [5] identified associations between liver injury markers and diabetes and used random forests to predict diabetes based on serum variables. Moreover, different techniques are sometimes combined, creating ensemble models to surpass the single model’s predictive performance.

The number of studies developed in the field creates two main challenges for researchers and developers aiming to build type 2 diabetes predictive models. First, there is considerable heterogeneity in previous studies regarding machine learning techniques used, making it challenging to identify the optimal one. Second, there is a lack of transparency about the features used to train the models, which reduces their interpretability, a feature utterly relevant to the doctor.

This review aims to inform the selection of machine learning techniques and features to create novel type 2 diabetes predictive models. The paper is organized as follows. “Background” section provides a brief background on the techniques used to create predictive models. “Methods” section presents the methods used to design and conduct the review. “Results” section summarizes the results, followed by their discussion in “Discussion” section, where a summary of findings, the opportunity areas, and the limitations of this review are presented. Finally, “Conclusions” section presents the conclusions and future work.

## Background

### Machine learning and deep learning

Over the last years, humanity has achieved technological breakthroughs in computer science, material science, biotechnology, genomics, and proteomics [6]. These disruptive technologies are shifting the paradigm of medical practice. In particular, artificial intelligence and big data are reshaping disease and patient management, shifting to personalized diagnosis and treatment. This shift enables public health to become predictive and preventive [6].

Machine learning is a subset of artificial intelligence that aims to create computer systems that discover patterns in training data to perform classification and prediction tasks on new data [7]. Machine learning puts together tools from statistics, data mining, and optimization to generate models.

Representational learning, a subarea of machine learning, focuses on automatically finding an accurate representation of the knowledge extracted from the data [7]. When this representation comprises many layers (i.e., a multi-level representation), we are dealing with deep learning.

In deep learning models, every layer represents a level of learned knowledge. The nearest to the input layer represents low-level details of the data, while the closest to the output layer represents a higher level of discrimination with more abstract concepts.

The studies included in this review used 18 different types of models:

- Deep Neural Network (DNN): DNNs are loosely inspired by the biological nervous system. Artificial neurons are simple functions depicted as nodes compartmentalized in layers, and synapses are the links between them [8]. DNN is a data-driven, self-adaptive learning technique that produces non-linear models capable of real-world modeling problems.
- Support Vector Machines (SVM): SVM is a non-parametric algorithm capable of solving regression and classification problems using linear and non-linear functions. These functions assign vectors of input features to an n-dimensional space called a feature space [9].
- k-Nearest Neighbors (KNN): KNN is a supervised, non-parametric algorithm based on the “things that look alike” idea. KNN can be applied to regression and classification tasks. The algorithm computes the closeness or similarity of new observations in the feature space to *k* training observations to produce their corresponding output value or class [9].
- Decision Tree (DT): DTs use a tree structure built by selecting thresholds for the input features [8]. This classifier aims to create a set of decision rules to predict the target class or value.
- Random Forest (RF): RFs merge several decision trees, such as bagging, to get the final result by a voting strategy [9].
- Gradient Boosting Tree (GBT) and Gradient Boost Machine (GBM): GBTs and GBMs join sequential tree models in an additive way to predict the results [9].
- J48 Decision Tree (J48): J48 develops a mapping tree to include attribute nodes linked by two or more sub-trees, leaves, or other decision nodes [10].
- Logistic and Stepwise Regression (LR): LR is a linear regression technique suitable for tasks where the dependent variable is binary [8]. The logistic model is used to estimate the probability of the response based on one or more predictors.
- Linear and Quadratic Discriminant Analysis (LDA): LDA segments an n-dimensional space into two or more dimensional spaces separated by a hyper-plane [8]. The aim of it is to find the principal function for every class. This function is displayed on the vectors that maximize the between-group variance and minimizes the within-group variance.
- Cox Hazard Regression (CHR): CHR or proportional hazards regression analyzes the effect of the features to occur a specific event [11]. The method is partially non-parametric since it only assumes that the effects of the predictor variables on the event are constant over time and additive on a scale.
- Least-Square Regression: (LSR) method is used to estimate the parameter of a linear regression model [12]. LSR estimators minimize the sum of the squared errors (a difference between observed values and predicted values).
- Multiple Instance Learning boosting (MIL): The boosting algorithm sequentially trains several weak classifiers and additively combines them by weighting each of them to make a strong classifier [13]. In MIL, the classifier is logistic regression.
- Bayesian Network (BN): BNs are graphs made up of nodes and directed line segments that prohibit cycles [14]. Each node represents a random variable and its probability distribution in each state. Each directed line segment represents the joint probability between nodes calculated using Bayes’ theorem.
- Latent Growth Mixture (LGM): LGM groups patients into an optimal number of growth trajectory clusters. Maximum likelihood is the approach to estimating missing data [15].
- Penalized Likelihood Methods: Penalizing is an approach to avoid problems in the stability of the estimated parameters when the probability is relatively flat, which makes it difficult to determine the maximum likelihood estimate using simple methods. Penalizing is also known as shrinkage [16]. Least absolute shrinkage and selection operator (LASSO), smoothed clipped absolute deviation (SCAD), and minimax concave penalized likelihood (MCP) are methods using this approach.
- Alternating Cluster and Classification (ACC): ACC assumes that the data have multiple hidden clusters in the positive class, while the negative class is drawn from a single distribution. For different clusters of the positive class, the discriminatory dimensions must be different and sparse relative to the negative class [17]. Clusters are like “local opponents” to the complete negative set, and therefore the “local limit” (classifier) has a smaller dimensional subspace than the feature vector.

Some studies used a combination of multiple machine learning techniques and are subsequently labeled as machine learning-based method (MLB).

### Systematic literature review methodologies

This review follows two methodologies for conducting systematic literature reviews: the Preferred Reporting Items for Systematic Reviews and Meta-Analyses (PRISMA) statement [18] and the Guidelines for performing Systematic Literature Reviews in Software Engineering [19]. Although these methodologies hold many similarities, there is a substantial difference between them. While the former was tailored for medical literature, the latter was adapted for reviews in computer science. Hence, since this review focuses on computer methods applied to medicine, both strategies were combined and implemented. The PRISMA statement is the standard for conducting reviews in the medical sciences and was the principal strategy for this review. It contains 27 items for evaluating included studies, out of which 23 are used in this review. The second methodology is an adaptation by Keele and Durham Universities to conduct systematic literature reviews in software engineering. The authors provide a list of guidelines to conduct the review. Two elements were adopted from this methodology. First, the protocol’s organization in three stages (planning, conducting, and reporting). Secondly, the quality assessment strategy to select studies based on the information retrieved by the search.

### Related works

Previous reviews have explored machine learning techniques in diabetes, yet with a substantially different focus. Sambyal et al. conducted a review on microvascular complications in diabetes (retinopathy, neuropathy, nephropathy) [20]. This review included 31 studies classified into three groups according to the methods used: statistical techniques, machine learning, and deep learning. The authors concluded that machine learning and deep learning models are more suited for big data scenarios. Also, they observed that the combination of models (ensemble models) produced improved performance.

Islam et al. conducted a review with meta-analysis on deep learning models to detect diabetic retinopathy (DR) in retinal fundus images [21]. This review included 23 studies, out of which 20 were also included for meta-analysis. For each study, the authors identified the model, the dataset, and the performance metrics and concluded that automated tools could perform DR screening.

Chaki et al. reviewed machine learning models in diabetes detection [22]. The review included 107 studies and classified them according to the model or classifier, the dataset, the features selection with four possible kinds of features, and their performance. The authors found that text, shape, and texture features produced better outcomes. Also, they found that DNNs and SVMs delivered better classification outcomes, followed by RFs.

Finally, Silva et al. [23] reviewed 27 studies, including 40 predictive models for diabetes. They extracted the technique used, the temporality of prediction, the risk of bias, and validation metrics. The objective was to prove whether machine learning exhibited discrimination ability to predict and diagnose type 2 diabetes. Although this ability was confirmed, the authors did not report which machine learning model produced the best results.

This review aims to find areas of opportunity and recommendations in the prediction of diabetes based on machine learning models. It also explores the optimal performance metrics, the datasets used to build the models, and the complementary techniques used to improve the model’s performance.

## Methods

### Objective of the review

This systematic review aims to identify and report the areas of opportunity for improving the prediction of diabetes type 2 using machine learning techniques.

### Research questions

1. Research Question 1 (RQ1): What kind of features make up the database to create the model?
2. Research Question 2 (RQ2): What machine learning technique is optimal to create a predictive model for type 2 diabetes?
3. Research Question 3 (RQ3): What are the optimal validation metrics to compare the models’ performance?

### Information sources

Two search engines were selected to search:

- PubMed, given the relationship between a medical problem such as diabetes and a possible computer science solution.
- Web of Science, given its extraordinary ability to select articles with high affinity with the search string.

These search engines were also considered because they search in many specialized databases (IEEE Xplore, Science Direct, Springer Link, PubMed Central, Plos One, among others) and allow searching using keywords combined with boolean operators. Likewise, the database should contain articles with different approaches to predictive models and not specialized in clinical aspects. Finally, the number of articles to be included in the systematic review should be sufficient to identify areas of opportunity for improving models’ development to predict diabetes.

### Search strategy

Three main keywords were selected from the research questions. These keywords were combined in strings as required by each database in their advanced search tool. In other words, these strings were adapted to meet the criteria of each database Table 1.

**Table 1:** Strings used in the search

Data Base	String of keywords
PubMed	((diabetes[Title] AND predictive) AND machine learning)
Web of Science	Ti=(diabetes) AND
	All=(predictive AND machine learning)

### Eligibility criteria

Retrieved records from the initial search were screened to check their compliance with eligibility criteria.

Firstly, papers published from 2017 to 2021 only were considered. Then, two rounds of screening were conducted. The first round focused mainly on the scope of the reported study. Articles were excluded if the study used genetic data to train the models, as this was not a type of data of interest in this review. Also, articles were excluded if the full text was not available. Finally, review articles were also excluded.

In the second round of screening, articles were excluded when machine learning techniques were not used to predict type 2 diabetes but other types of diabetes, treatments, or diseases associated with diabetes (complications and related diseases associated with metabolic syndrome). Also, studies using unsupervised learning were excluded as they cannot be validated using the same performance metrics as supervised learning models, preventing comparison.

### Quality assessment

After retrieving the selected articles, three parameters were selected, each one generated by each research question. The eligibility criteria are three possible subgroups according to the extent to which the article satisfied it.

1. The dataset contains sociodemographic and lifestyle data, clinical diagnosis, and laboratory test results as attributes for the model.
  1.1. Dataset contains only one kind of attributes.
  1.2. Dataset contains similar kinds of attributes.
  1.3. Dataset uses EHRs with multiple kinds of attributes.
2. The article presents a model with a machine learning technique to predict type 2 diabetes.
  2.1. Machine Learning methods are not used at all.
  2.2. The prediction method in the model is used as part of the prepossessing for the data to do data mining.
  2.3. Model used a machine learning technique to predict type 2 diabetes.
3. The authors use supervised learning with validation metrics to contrast their results with previous work.
  3.1. The authors used unsupervised methods.
  3.2. The authors used a supervised method with one validation metric or several methods with supervised and unsupervised learning.
  3.3. The authors used supervised learning with more than one metric to validate the model (accuracy, specificity, sensitivity, area under the ROC, F1-score).

### Data extraction

After assessing the papers for quality, the intersection of the subgroups QA2.3 and QA1.1 or QA1.2 or QA1.3 and QA3.2 or QA3.3 were processed as follows.

First, the selected articles were grouped in two possible ways according to the data type (glucose forecasting or electronic health records). The first group contains models that screen the control levels of blood glucose, while the second group contains models that predict diabetes based on electronic health records.

The second classification was more detailed, applying for each group the below criteria.

The data extraction criteria are:

- Machine learning model (specify which machine learning method use)
- Validation parameter (accuracy, sensitivity, specificity, F1-score, AUC (ROC))
- Complementary techniques (complementary statistics and machine learning techniques used for the models)
- Data sampling (cross-validation, training-test set, complete data)
- Description of the population (age, balanced or imbalance, population cohort size).

### Risk of bias analyses

#### Risk of bias in individual studies

The risk of bias in individual studies (i.e., within-study bias) was assessed based on the characteristics of the sample included in the study and the dataset used to train and test the models. One of the most common risks of bias is when the data is imbalanced. When the dataset has significantly more observations for one label, the probability of selecting that label increases, leading to misclassification.

The second parameter that causes a risk of bias is the age of participants. In most cases, diabetes onset would be in older people making possible bound between 40 to 80 years. In other cases, the onset occurs at early age generating another dataset with a range from 21 to 80.

A third parameter strongly related to age is the early age onset. Complications increase and appear early when a patient lives more time with the disease, making it harder to develop a model only for diabetes without correlation of their complications.

Finally, as the fourth risk of bias, according to Forbes [24] data scientists spend 80% of their time on data preparation, and 60% of it is in data cleaning and organization. A well-structured dataset is relevant to generate a good performance of the model. That can be check in the results from the data items extraction the datasets like PIMA dataset that is already clean and organized well generate a model with the recall of 1 [25] also the same dataset reach an accuracy of 0.97 [26] in another model. Dirty data can not achieve values as good as clean data.

#### Risk of bias across studies

The items considered to assess the risk of bias across the studies (i.e., between-study bias) were the reported validation parameters and the dataset and complementary techniques used.

Validation metrics were chosen as they are used to compare the performance of the model. The studies must be compared using the same metrics to avoid bias from the validation methods.

The complementary techniques are essential since they can be combined with the primary approach to creating a better performance model. It causes a bias because it is impossible to discern if the combination of the complementary and the machine learning techniques produces good performance or if the machine learning technique per se is superior to others.

## Results

### Search results and reduction

The initial search generated 1327 records, 925 from PubMed and 402 from Web of Science. Only 130 records were excluded when filtering by publication year (2017–2021). Therefore, further searches were conducted using fine-tuned search strings and options for both databases to narrow down the results. The new search was carried out using the original keywords but restricting the word ‘diabetes’ to be in the title, which generated 517 records from both databases. Fifty-one duplicates were discarded. Therefore, 336 records were selected for further screening.

Further selection was conducted by applying the exclusion criteria to the 336 records above. Thirty-seven records were excluded since the study reported used non-omittable genetic attributes as model inputs, something out of this review’s scope. Thirty-eight records were excluded as they were review papers. All in all, 261 articles that fulfill the criteria were included in the quality assessment.

**Fig. 1:**
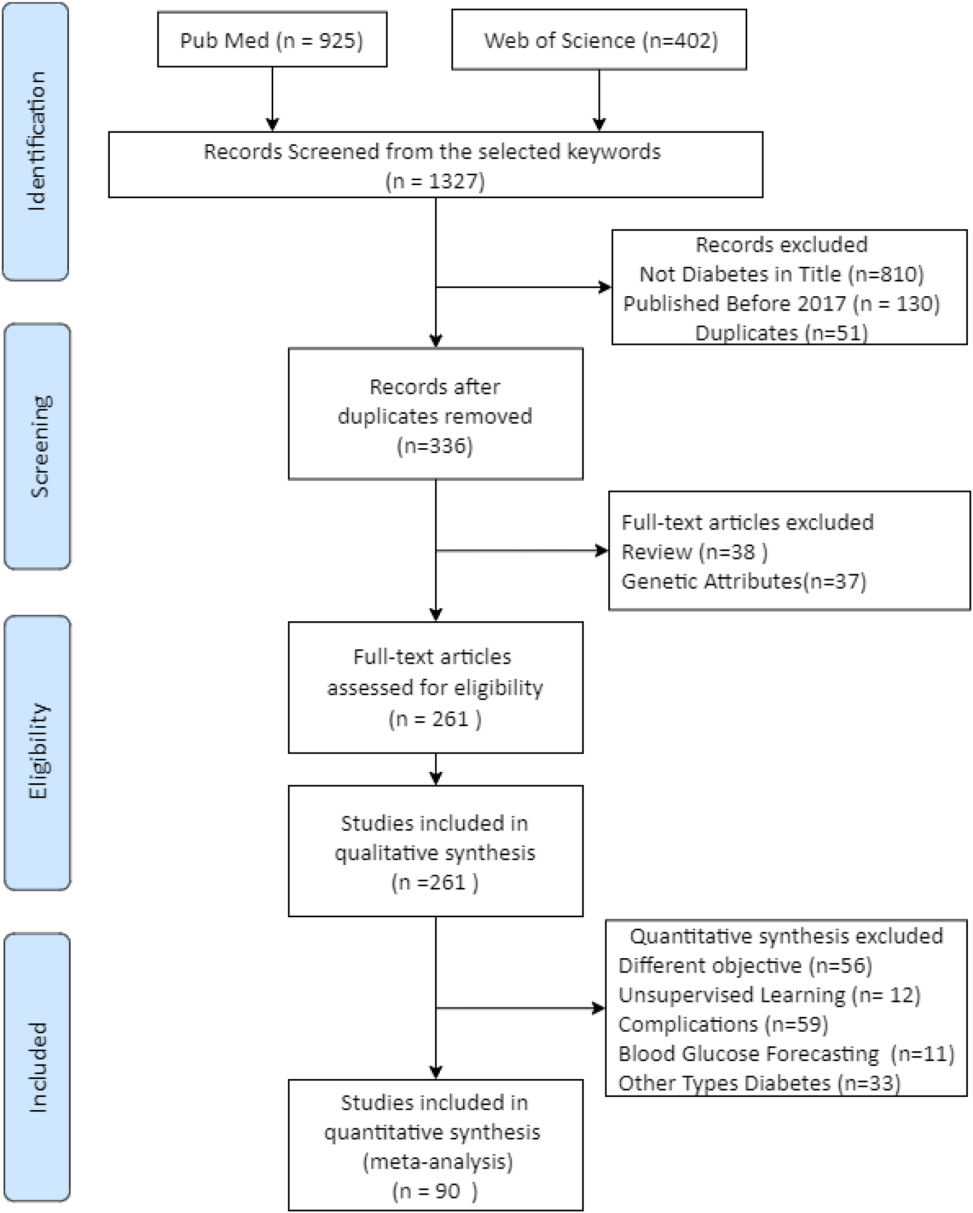
Flow diagram indicating the results of the systematic review with inclusions and exclusions

Figure 1 shows the flow diagram summarizing this process.

### Quality assessment

**Fig. 2:**
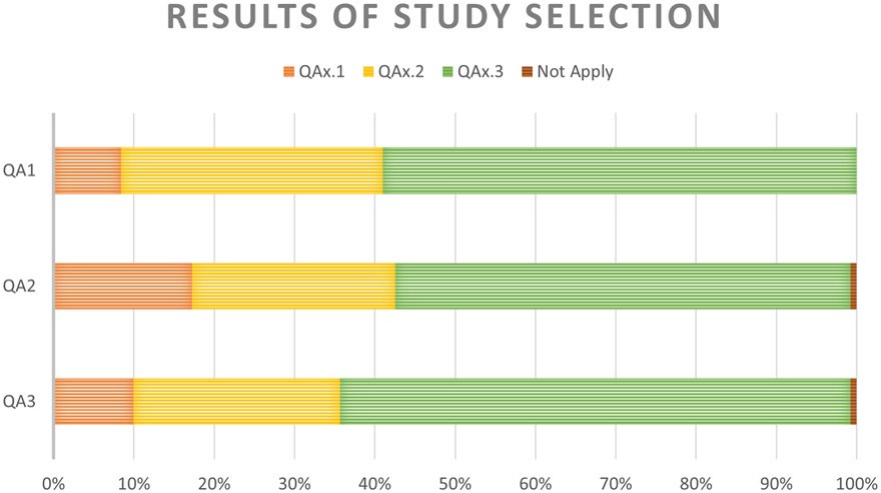
Percentage of each subgroup in the quality assessment. The criteria does not apply for two result for the Quality Assessment Questions 1 and 3

The 261 articles above were assessed for quality and classified into their corresponding subgroup for each quality question (Fig. 2).

The first question classified the studies by the type of database used for building the models. The third subgroup represents the most desirable scenario. It includes studies where models were trained using features from Electronic Health Records or a mix of datasets including lifestyle, socio-demographic, and health diagnosis features. There were 22, 85, and 154 articles in subgroups one to three, respectively.

The second question classified the studies by the type of model used. Again, the third subgroup represents the most suitable subgroup as it contains studies where a machine learning model was used to predict diabetes onset. There were 46 studies in subgroup one, 66 in subgroup two, and 147 in subgroup three. Two studies were omitted from these subgroups: one used cancer-related model; another used a model of no interest to this review.

The third question clustered the studies based on their validation metrics. There were 25 studies in subgroup one (semi-supervised learning), 68 in subgroup two (only one validation metric), and 166 in subgroup three ($>1$ validation parameters). The criteria are not applied to two studies as they used special error metrics, making it impossible to compare their models with the rest.

Data extraction excluded 101 articles from the quantitative synthesis for two reasons. twelve studies used unsupervised learning. Nineteen studies focused on diabetes treatments, 33 in other types of diabetes (eighteen type 1 and fifteen Gestational), and 37 associated diseases.

Furthermore, 70 articles were left out of this review as they focus on the prediction of diabetes complications (59) or tried to forecast levels of glucose (11), not onset. Therefore, 90 articles were chosen for the next steps.

### Data extraction

Table 2 summarize the results of the data extraction. These tables are divided into two main groups, each of them corresponding to a type of data.

**Table 2:** Detailed classification of methods that predict the main factors for diagnosing the onset of diabetes

Cite	References	Machine learning model	Validation parameter	Data sampling	Complementary techniques	Description of the population
*Type of data: electronic health records*						
[29]	Arellano-Campos et al. (2019)	Cox proportional hazard regression	Accuracy: 0.75 hazard ratios	Cross-validation (k = 10) and bootstrapping	Beta-coefficients model	Base L: 7636 follow: 6144 diabetes: 331 age: 32–54
[30]	You et al. (2019)	Super learning: ensemble learner by choosing a weighted combination of algorithms	Average treatment effect	Cross-validation	Targeted learning query language logistic and tree regression	Total: 78,894 control: 41,127 diabetes: 37,767 age: > 40
[27]	Maxwell et al. (2017)	Sigmoid function-Deep Neural Network with cross entropy as loss function	Accuracy: 0.921 F1-score: 0.823 precision: 0.915 sensitivity: 0.867	Training set (90%) test set (10%) tenfold cross-validation	RAkEL-LibSVM RAkEL-MLP RAkEL-SMO RAkEL-J48 RAkEL-RF MLkNN	Total: 110,300 imbalanced 6 disease categories
[28]	Nguyen et al. (2019)	Deep Neural Network with three embedding and two hidden layers	Specificity 0.96 accuracy: 0.84 sensitivity: 0.31 AUC (ROC): 0.84	Training set (70%): cross-validation 9:1 test set (30%)	Generalized linear model large-scale regression	Total: 76,214 78 diseases age: 25–78
[31]	Pham et al. (2017)	Recurrent Neural Network Convolutional-Long Short-Term Memory (C-LSTM)	F1-score: 0.79 precision: 0.66	Training set (66%) tuning set (17%) test set (17%)	Support vector machine and random forests	Diabetes: 12,000 age: 18–100 mean age: 73
[32]	Spänig et al. (2019)	Deep Neural Networks with tangens hyperbolicus	AUC (ROC) = 0.71 AUC (ROC) = 0.68	Training set (80%) test set (20%)	Sub-sampling approach support vector machine with RBF kernel	Total: 4814 diabetes: 646 diagnosis: 397 not diag: 257 age: 45–75 imbalance
[33]	Wang et al. (2020)	Convolutional neural network and bidirectional long short-term memory	Precision: 92.3 recall: 90.5 F score: 91.3 accuracy: 92.8	Training set (70%) validation set (10%) test set (20%)	SVM-TFIDF CNN BiLSTM	Total: 18,625 diabetes: 5645 10 disease categories
[34]	Kim et al. (2020)	Class activation map and CNN (SSANet)	R2 = 0.75 MAE = 3.55 AUC (ROC) = 0.77	Training set (89%) validation set (1%) test set (10%)	Linear regression	Total: 412,026 norm: 243,668 diabetes: 14,189 age: 19–90
[35]	Bernardini et al. (2020)	Sparse balanced support vector machine (SB-SVM)	Recall = 0.7464 AUC (ROC) = 0.8143	Tenfold cross-validation	Sparse 1-norm SVM	Total: 2433 diabetes: 225 control: 2208 age: 60–80 imbalanced
[36]	Mei et al. (2017)	Hierarchical recurrent neural network	AUC (ROC) = 0.9268 Accuracy = 0.6745	Training set (80%) validation set (10%) test set (10%)	Linear regression	Total: 620,633
[25]	Prabhu et al. (2019)	Deep belief neural network	Recall: 1.0 precision: 0.68 F1 score: 0.80	Training set validation set test set	Principal component analysis	Pima Indian Women Diabetes Dataset
[13]	Bernardini et al. (2020)	Multiple instance learning boosting	Accuracy: 0.83 F1-score: 0.81 precision: 0.82 recall: 0.83 AUC (ROC): 0.89	Tenfold cross-validation	None	Total: 252 diabetes: 252 age: 54–72
[37]	Solares et al. (2019)	Hazard ratios using Cox regression	AUC (ROC): 0.75, concordance (C-statistic)	Derivation set (80%) validation (20%)	None	Total: 80,964 diabetes: 2267 age: 50
[38]	Kumar et al. (2017)	Support vector machine, Naive Bayes, K-nearest neighbor C4.5 decision tree	Precision: 0.65, 0.68, 0.7, 0.72 recall: 0.69, 0.68, 0.7, 0.74 accuracy: 0.69, 0.67, 0.7, 0.74 F-score: 0.65, 0.68, 0.7, 0.72	N-fold (N = 10) cross validation	None	Diabetes: 200 age: 1–100
[39]	Olivera et al. (2017)	Logistic regression artificial neural network K-nearest neighbor Naïve Bayes	AUC (ROC): 75.44, 75.48, 74.94, 74.47 balanced accuracy: 69.3, 69.47, 68.74, 68.95	Training set (70%) test set (30%) tenfold cross-validation	Forward selection	Diabetes: 12,447 unknown: 1359 age: 35–74
[10]	Alghamdi et al. (2017)	Naïve Bayes tree, random forest, and logistic model tree, j48 decision tree	Kappa: 1.34, 3.63 1.37, 0.70, 1.14 recall (%) 99.2, 99.2, 90.8, 99.9, 99.4 Specificity (%) 1.6, 3.1, 21.2 0.50, 1.3 accuracy (%) 83.9, 84.1, 79.9, 84.3, 84.1	N-fold cross validation	Multiple linear regression gain ranking method synthetic minority oversampling technique	Total: 32,555 diabetes: 5099 imbalanced
[14]	Xie et al. (2017)	K2 structure-learning algorithm	Accuracy = 82.48	Training set (75%) test set (25%)	None	Total: 21,285 diabetes: 1124 age: 35–65
[40]	Peddinti et al. (2017)	Regularised least-squares regression for binary risk classification	Odds ratio accuracy: 0.77	Tenfold cross-validation	Logistic regression	Total: 543 diabetes: 146 age: 48–50
[8]	Maniruzzaman et al. (2017)	Linear discriminant analysis, quadratic discriminant analysis, Naïve Bayes, Gaussian process classification, support vector machine, artificial neural network, Adaboost, logistic regression, decision tree, random forest	Accuracy: 0.92 sensitivity: 0.96 specificity: 0.80 PPV: 0.91 NPV: 0.91 AUC (ROC): 0.93	Cross-validation K2, K4, K5, K10, and JK	Random forest, logistic regression, mutual information, principal component analysis, analysis of variance Fisher discriminant ratio	Pima Indian diabetic dataset
[41]	Dutta et al. (2018)	Logistic regression support vector machine random forest	Sensitivity: 0.80, 0.75, 0.84 F1-score: 0.80, 0.79, 0.84	Training set (67%) test set (33%)	None	Diabetes: 130 control: 262 imbalanced age: 21–81
[42]	Alhassan et al. (2018)	Long short-term memory deep learning gated-recurrent unit deep learning	Accuracy: 0.97 F1-score: 0.96	Training set (90%) test set (10%) tenfolds cross-validation	Logistic regression support vector machine, multi-layer perceptron	Total: 41,000,000 imbalanced diabetes: 62%
[15]	Hertroijs et al. (2018)	Latent growth mixture modelling	Specificity: 81.2% sensitivity: 78.4% accuracy: 92.3%	Training set (90%) test set (10%) fivefold cross-validation	K-nearest neighbour	Total: 105814 age: > 18
[43]	Kuo et al. (2020)	Random forest C5.0 support vector machine	Accuracy: 1 F1-score: 1 AUC (ROC): 1 sensitivity: 1	Tenfold cross-validation	Information gain (features) gain ratio	Total: 149 diabetes: 149 age: 21–91
[44]	Pimentel et al. (2018)	Naïve Bayes, alternating decision tree, random forest, random tree, k-nearest neighbor, support vector machine	Specificity: 0.76, 0.88, 0.87, 0.97, 0.82, 0.85 sensitivity: 0.62, 0.50, 0.33, 0.42, 0.40, 0.59 AUC (ROC): 0.73, 0.81, 0.87, 0.74, 0.62, 0.63	Training set (70%) test set (30%) tenfold cross-validation	SMOTE	Total: 9947 imbalanced diabetes: 13% age: 21–93
[45]	Talaei-Khoeni et al. (2018)	Artificial neural network, support vector machine, logistic regression, decision tree	AUC (ROC): 0.614, 0.831, 0.738, 0.793 sensitivity: 0.608, 0.683, 0.677, 0.687 specificity: 0.783, 0.950, 0.712, 0.651 MCC: 0.797. 0.922, 0.581, 0.120 MCE: 0.844, 0.989, 0.771, 0.507	Oversampling technique, random under sampling	Syntactic minority LASSO, AIC and BIC	Total: 10,911 imbalance diabetes: 51.9%
[46]	Perveen et al. (2019)	J48 decision tree, Naïve Bayes	TPR: 0.85, 0.782, 0.852, 0.774 FPR: 0.218, 0.15 0.226, 0.148 precision: 0.814, 0.782, 0.807 recall: 0.85, 0.802, 0.852, 0.824 F-measure: 0.831, 0.634, 0.829, 0.774 MCC: 0.634, 0.823, 0.628, 0.798 AUC (ROC): 0.883, 0.873, 0.836, 0.826	K-medoids under sampling	Logistic regression	Total: 667, 907 age: 22–74 diabetes: 8.13% imbalance
[47]	Yuvaraj et al. (2019)	Decision tree Naïve Bayes random forest	Precision: 87, 91, 94 recall: 77, 82, 88 F-measure: 82, 86, 91 accuracy: 88, 91, 94	Training set (70%) test set (30%)	Information gain RHadoop	Total: 75,664
[48]	Deo et al. (2019)	Bagged trees, linear support vector machine	Accuracy: 91% AUC (ROC): 0.908	Training set (70%) test set (30%) fivefold cross-validation, holdout validation	Synthetic minority oversampling technique, Gower’s distance	Total: 140 diabetes: 14 imbalanced age: 12–90
[49]	Jakka et al. (2019)	K nearest neighbor, decision tree, Naive Bayes, support vector machine, logistic regression, random forest	Accuracy: 0.73, 0.70, 075, 0.66, 0.78, 0.74 recall: 0.69, 0.72, 0.74, 0.64 0.76, 0.69 F1-score: 0.69, 0.72, 0.74, 0.40, 0.75, 0.69 misclassification rate: 0.31, 0.29, 0.26, 0.36, 0.24, 0.29 AUC (ROC): 0.70, 0.69, 0.70, 0.61, 0.74, 0.70	None	None	Pima Indians Diabetes dataset
[50]	Radja et al. (2019)	Naive Bayes, support vector machine, decision table, J48 decision tree	Precision: 0.80, 0.79, 0.76, 0.79 precision: 0.68, 0.74, 0.60, 0.63 recall: 0.84, 0.90, 0.81, 0.81 recall: 0.61, 0.54, 0.53, 0.60 F1-score: 0.76, 0.76, 0.71, 0.74	Tenfold cross-validation	None	Total: 768 diabetes: 500 control: 268
[51]	Choi et al. (2019)	Logistic regression, linear discriminant analysis, quadratic discriminant analysis, K-nearest neighbor	AUC (ROC): 0.78, 0.77 0.76, 0.77	Tenfold cross-validation	Information gain	Total: 8454 diabetes: 404 age: 40–72
[52]	Akula et al. (2019)	K nearest neighbor, support vector machine, decision tree, random forest, gradient boosting, neural network, Naive Bayes	Overall accuracy: 0.86 precision: 0.24 negative prediction: 0.99 sensitivity: 0.88 specificity: 0.85 F1-score: 0.38	Training set: 800 test set: 10,000	None	Pima Indians Diabetes Dataset Practice Fusion Dataset total: 10,000 age: 18–80
[53]	Xie et al. (2019)	Support vector machine, decision tree, logistic regression, random forest, neural network, Naive Bayes	Accuracy: 0.81, 0.74, 0.81, 0.79, 0.82, 0.78 sensitivity: 0.43, 0.52, 0.46, 0.50, 0.37, 0.48 specificity: 0.87, 0.78, 0.87, 0.84 0.90, 0.82 AUC (ROC): 0.78, 0.72, 0.79, 0.76, 0.80, 0.76	Training set (67%) test set (33%)	Odds ratio synthetic minority over-sampling technique	Total: 138,146 diabetes: 20,467 age: 30–80
[54]	Lai et al. (2019)	Gradient boosting machine, logistic regression, random forest, Rpart	AUC (ROC): 84.7%, 84.0% 83.4%, 78.2%	Training set (80%) test set (20%) tenfold cross-validation	Misclassification costs	Total: 13,309 diabetes: 20.9% age: 18–90 imbalanced
[17]	Brisimi et al. (2018)	Alternating clustering and classification	AUC (ROC): 0.8814, 0.8861, 0.8829, 0.8812	Training set (40%) test set (60%)	Sparse (l1-regularized), support vector machines, random forests, gradient tree boosting	Diabetes: 47,452 control: 116,934 age mean: 66
[55]	Abbas et al. (2019)	Support vector machine with Gaussian radial basis	Accuracy: 96.80% sensitivity: 80.09%	Tenfold cross-validation	Minimum redundancy maximum relevance algorithm	Total: 1438 diabetes: 161 age: 25–64
[56]	Sarker et al. (2020)	K-nearest neighbors	Precision: 0.75 recall: 0.76 F-score: 0.75 AUC (ROC): 0.72	Tenfold cross validation	Adaptive boosting, logistic regression, Naive Bayes, support vector machine decision tree	Total: 500 age: 10–80
[57]	Cahn et al. (2020)	Gradient boosting trees model	AUC (ROC): 0.87 sensitivity: 0.61 specificity: 0.91 PPV: 0.16	Training set: THIN dataset validation set: AppleTree dataset MHS dataset	Logistic-regression	Age: 40–80 THIN: total = 3,068,319 pre-DM: 40% DM: 2.9% Apple Tree: P-DM: 381,872 DM: 2.3% MHS: pre-DM: 12,951 DM: 2.7%
[58]	Garcia-Carretero et al. (2020)	K-nearest neighbors	Accuracy: 0.977 sensitivity 0.998 specificity 0.838 PPV: 0.976 NPV: 0.984 AUC (ROC): 0.89	Tenfold cross-validation	Random forest	Age: 44–72 pre-DM = 1647 diabetes: 13%
[59]	Zhang et al. (2020)	Logistic regression, classification and regression tree, gradient boosting machine, artificial neural networks, random forest, support vector machine	AUC (ROC): 0.84, 0.81, 0.87, 0.85, 0.87, 0.84 accuracy: 0.75, 0.80, 0.81, 0.74, 0.86, 0.76 sensitivity: 0.79, 0.67, 0.76, 0.81, 0.80, 0.75 specificity: 0.75, 0.81, 0.82, 0.73, 0.78, 0.77 PPV: 0.23, 0.26, 0.29, 0.26, 0.26, 0.24 NPV: 0.97, 0.96, 0.97, 0.98, 0.98, 0.97	Tenfold cross-validation	Synthetic minority over-sampling technique	Total: 36,652 age: 18–79
[26]	Albahli et al. (2020)	Logistic regression	Accuracy: 0.97	Tenfold cross-validation	Random Forest eXtreme Gradient Boosting	Total: diabetes age: 21–81 Pima Indians Diabetes dataset
[60]	Haq et al. (2020)	Decision tree (iterative Dichotomiser 3)	Accuracy: 0.99 sensitivity 1 specificity 0.98 MCC: 0.99 F1-score: 1 AUC (ROC): 0.998	Training set (70%) test set (30%) hold out training set (90%) test set (10%) tenfold cross-validation	Ada Boost, random forest	Total = 2000 diabetes: 684 age: 21–81
[61]	Yang et al. (2020)	Linear discriminant analysis, support vector machine random forest	AUC: 0.85, 0.84, 0.83 sensitivity: 0.80, 0.79, 0.78 specificity: 0.74, 0.75, 0.73 accuracy: 0.75 0.74,0.74 PPV: 0.36, 0.36, 0.35	Training set: (80%, 2011–2014), test set: (20%, 2011–2014) and validation set: (2015–2016) fivefold cross-validation	Binary logistic regression	Total = 8057 age: 20–89 imbalanced
[62]	Ahn et al. (2020)	Random forest, support vector machine	AUC (ROC): 1.00, 0.95	Tenfold cross-validation	ELISA	Age: 43–68
[63]	Sarwar et al. (2018)	K nearest neighbors, Naive Bayes, support vector machine, decision tree, logistic regression, random forest	Accuracy: 0.77, 0.74, 0.77, 0.71, 0.74, 0.71	Training set (70%) test set (30%) tenfold cross-validation	None	Pima Indians Diabetes Dataset
[64]	Zou et al. (2018)	Random forest J48 decision tree Deep Neural Network	Accuracy: 0.81, 0.79, 0.78 sensitivity: 0.85 0.82, 0.82 specificity: 0.77, 0.76, 0.75 MCC: 0.62, 0.57, 0.57	Fivefold cross-validation	Principal component analysis, minimum redundancy maximum relevance	Pima Indian diabetic Luzhou
[65]	Farran et al. (2019)	Logistic regression k-nearest neighbours support vector machine	AUC (ROC): 3-year: 0.74, 0.83, 0.73 5-year: 0.72, 0.82, 0.68 7-year: 0.70, 0.79, 0.71	Fivefold cross-validation	None	Diabetes: 40,773 control: 107,821 age: 13–65
[66]	Xiong et al. (2019)	Multilayer perceptron, AdaBoost, random forest, support vector machine, gradient boosting	Accuracy: 0.87, 0.86, 0.86, 0.86, 0.86	Training set (60%) test set (20%) tenfolds cross-validation set (20%)	Missing values feature mean	Total: 11845 diabetes: 845 age: 20–100
[67]	Dinh et al. (2019)	Support vector machine, random forest, gradient boosting, logistic regression	AUC (ROC): 0.890.94, 0.96, 0.72 sensitivity: 0.81, 0.86, 0.89, 0.67 precision: 0.81, 0.86, 0.89, 0.67 F1-score: 0.81, 0.86, 0.89, 0.67	Training set (80%) test set (20%) tenfold cross-validation	None	Case 1: 21,131 diabetes: 5532 case 2: 16,426 prediabetes: 6482
[68]	Liu et al. (2019)	LASSO, SCAD, MCP, stepwise regression	AUC (ROC): 0.710.70, 0.70, 0.71 sensitivity: 0.64, 0.64, 0.64, 0.63 specificity: 0.68, 0.68, 0.68, 0.68, precision: 0.35, 0.35, 0.35, 0.35 NPV: 0.87, 0.87, 0.87, 0.87	Training set (70%) test set (30%) tenfold cross-validation	None	Total: 5481 age: > 40
[9]	Muhammad et al. (2020)	Logistic regression support vector machine K-nearest neighbor random forest Naive Bayes gradient boosting	Accuracy: 0.81, 0.85, 0.82, 0.89, 0.77, 0.86 AUC (ROC): 0.80, 0.85, 0.82, 0.86 0.77, 0.86	None	Correlation coefficient analysis	Total: 383 age: 1–150 diabetes: 51.9%
[69]	Tang et al. (2020)	EMR-image multimodal network (CNN)	Accuracy: 0.86 F1-score: 0.76 AUC (ROC): 0.89 Sensitivity: 0.68 Precision: 0.88	Fivefold cross-validation	None	Total: 997 diabetes: 401
[70]	Maniruzzaman et al. (2021)	Naive Bayes decision tree Adaboost random forest	Accuracy: 0.87, 0.90, 0.91, 0.93 AUC (ROC): 0.82, 0.78, 0.90, 0.95	Tenfold cross-validation	Logistic regression	Total: 6561 diabetes: 657 age: 30–64 imbalanced
[71]	Boutilier et al. (2021)	Random forest logistic regression Adaboost K-nearest neighbors decision trees	AUC (ROC): 0.91, 0.91, 0.90, 0.86, 0.78	Tenfold cross-validation	2-Sided Wilcoxon signed rank test	Total: 2278 diabetes: 833 age: 35–63
[72]	Li et al. (2021)	Extreme gradient boosting (GBT)	AUC (ROC): 0.91 precision: 0.82 sensitivity: 0.80 F1-score: 0.77	Training set (60%) validation (20%) test set (20%)	Genetic algorithm	Diabetics: 570 control: 570 prediabetics: 570 age: 33–68
[73]	Lam et al. (2021)	Random forest logistic regression extreme gradient boosting GBT	AUC (ROC): 0.86 F1-score: 0.82	Tenfold cross-validation	None	Control: 19,852 diabetes: 3103 age: 40–69
[74]	Deberneh et al. (2021)	Random forest support vector machine XGBoost	Accuracy: 0.73, 0.73, 0.72 precision: 0.74, 0.74, 0.74 F1-score: 0.74, 0.74, 0.73 sensitivity: 0.73, 0.74, 0.72 Kappa: 0.60, 0.60, 0.58 MCC: 0.60, 0.60, 0.58	Tenfold cross-validation	ANOVA, Chi-squared, SMOTE feature Importance	Total: 535,169 diabetes: 4.3% prediabetes: 36% age: 18–108
[75]	He et al. (2021)	Cox regression	C-statics: 0.762	Hold out	None	Total: 68,299 diabetes: 1281 age: 40–69
[76]	García-Ordás et al. (2021)	Convolutional neural network (DNN)	Accuracy: 0.92	Training set (90%) test set (10%)	Variational and sparse autoencoders	Pima Indians
[77]	Kanimozhi et al. (2021)	Hybrid particle swarm optimization-artificial fish swarm optimization	Accuracy: 1, 0.99 specificity: 0.86, 0.83 sensitivity: 1, 0.99 MCC: 0.91, 0.92 Kappa: 0.96, 0.98	Training set (90%) test set (10%) fivefold cross-validation	Min–max scaling, kernel extreme learning machine	Pima Indians Diabetics, Diabetic Research Center
[78]	Ravaut et al. (2021)	Extreme gradient boosting tree	AUC (ROC): 0.84	Training set (86%) validation (7%) test set (7%)	Mean absolute Shapley values	Total: 15,862,818 diabetes: 19,137 age: 40–69
[79]	De Silva et al. (2021)	Logistic regression	AUC (ROC): 0.75 accuracy: 0.62 specificity: 0.62 sensitivity: 0.77 PPV: 0.09 NPV: 0.98	Training set (30%) validation (30%) test set (40%)	SMOTE ROSE	Total: 16,429 diabetes: 5.6% age: >20
[80]	Kim et al. (2021)	Deep neural network, logistic regression, decision tree	Accuracy: 0.80, 0.80, 0.71	Fivefold cross-validation	Wald test	Total: 3889 diabetes: 746 age: 40–69
[81]	Vangeepuram et al. (2021)	Naive Bayes	AUC (ROC): 0.75 accuracy: 0.62 specificity: 0.62 sensitivity: 0.77 PPV: 0.09 NPV: 0.98	Fivefold cross-validation	Friedman-Nemenyi	Total: 2858 diabetes: 828 age: 12–19
[82]	Recenti et al. (2021)	Random forest Ada-boost gradient boosting	Accuracy: 0.90, 0.79, 0.86 precision: 0.88, 0.78, 0.84 F1-score: 0.90, 0.81, 0.87 sensitivity: 0.93, 0.84, 0.90 specificity: 0.87, 0.76, 0.82 AUC (ROC): 0.97, 0.90, 0.95	Tenfold cross-validation	SMOTE	Total: 2943 age: 66–98 imbalance
[83]	Ramesh et al. (2021)	Support vector machine	Accuracy: 0.83 specificity: 0.79 sensitivity: 0.87	Tenfold cross-validation	MICE LASSO	Pima Indians
[84]	Lama et al. (2021)	Random forest	AUC (ROC): 0.78	Fivefold cross-validation	SHAP TreeExplainer	Total: 3342 diabetes: 556 age: 35–54
[85]	Shashikant et al. (2021)	Gaussian process-based kernel	Accuracy: 0.93 precision: 0.94 F1-score: 0.95 sensitivity: 0.96 specificity: 0.82 AUC (ROC): 0.89	Tenfold cross-validation	Non-linear HRV	Total: 135 diabetes: 100 age: 20–70
[86]	Kalagotla et al. (2021)	Stacking multi-layer perceptron, support vector machine logistic regression	Accuracy: 0.78 precision: 0.72 sensitivity: 0.51 F1-score: 0.60	Hold out k-fold cross-validation	Matrix correlation	Pima Indians
[87]	Moon et al. (2021)	Logistic regression	AUC (ROC): 0.94	Training set (47%) validation (30%) test set (23%)	Cox regression	Total: 14,977 diabetes: 636 age: 48–69
[88]	Ihnaini et al. (2021)	Ensemble deep learning model	Accuracy: 0.99 precision: 1 sensitivity: 0.99 F1-score: 0.99 RMSE: 0 MAE: 0.6	Hold out	None	Pima Indians merged Hospital Frenkfurt Germany
[89]	Rufo et al. (2021)	LightGBM	Accuracy: 0.98 specificity: 0.96 AUC (ROC): 0.98 Sensitivity: 0.99	Tenfold cross-validation	Min–max scale	Diabetes: 1030 Control: 1079 age: 12–90
[90]	Haneef et al. (2021)	Linear discriminant analysis	Accuracy: 0.67 specificity: 0.67 sensitivity: 0.62	Training set (80%) test set (20%)	Z-score transformation random down sampling	Total 44,659 age 18–69 imbalanced
[91]	Wei et al. (2022)	Random forest	AUC (ROC): 0.70 R2: 0.40	Training set (70%) test set (30%) tenfold cross-validation	LASSO PCA	Total: 8501 age: 15–50 diabetes: 8.92% imbalanced
[92]	Leerojanaprapa et al. (2019)	Bayesian network	AUC (ROC): 0.78	Training set (70%) test set (30%)	None	Total: 11,240 diabetes: 5.53% age: 15–19
[93]	Subbaiah et al. (2020)	Random forest	Accuracy: 1 specificity: 1 sensitivity: 1 Kappa: 1	Training set (70%) test set (30%)	None	Pima Indians
[94]	Thenappan et al. (2020)	Support vector machine	Accuracy: 0.97 specificity: 0.96 sensitivity: 0.94 precision: 0.96	Training set (70%) test set (30%)	Principal component analysis	Pima Indians
[95]	Sneha et al. (2019)	Support vector machine, random forest, Naive Bayes, decision tree, k-nearest neighbors	Accuracy: 0.78, 0.75, 0.74, 0.73, 0.63	Training set (70%) test set (30%)	None	Total: 2500 age: 29–70
[96]	Jain et al. (2020)	Support vector machine, random forest, k-nearest neighbors	Accuracy: 0.74 0.74, 0.76 precision: 0.67, 0.72, 0.70 sensitivity: 0.52, 0.44, 0.54 F1-score: 0.58, 0.55, 0.61 AUC (ROC): 0.74, 0.83, 0.83	Training set (70%) test set (30%)	None	Control: 500 diabetes: 268 age: 21–81
[97]	Syed et al. (2020)	Decision forest	F1-Score: 0.87 precision: 0.81 AUC (ROC): 0.90 Sensitivity: 0.91	Training set (80%) test set (20%)	Pearson Chi-squared	Total: 4896 diabetes: 990 age: 40–60
[98]	Nuankaew et al. (2020)	Average weighted objective distance	Precision: 0.99 accuracy: 0.90 specificity: 0.97	Training set (70%) test set (30%)	None	Mendeley data for diabetes
[99]	Samreen et al. (2021)	Stack NB, LR, KNN, DT, SVM, RF, Ada-boost, GBT	Accuracy: 0.98, 0.99 (SVD)	Training set (70%) test set (30%) tenfold cross-validation	One hot encoding, singular value decomposition	Age: 20–90
[100]	Fazakis et al. (2021)	Weighted voting LR-RF	AUC (ROC): 0.88	Hold-out	Forward/backward stepwise selection	English longitudinal study of ageing
[101]	Omana et al. (2021)	Newton’s divide difference method	Accuracy: 0.97 S-error: 0.06	Hold-out	Non-linear autoaggressive regression	Total: 812,007 diabetes: 23.49%
[102]	Ravaut et al. (2021)	Extreme gradient boosting tree	AUC (ROC): 0.80	Training set (87%) validation (7%) test set (6%)	Mean absolute Shapley values	Total: 14,786,763 diabetes: 27,820 age: 10–100 imbalance
[103]	Lang et al. (2021)	Deep belief network	AUC (ROC): 0.82 sensitivity: 0.80 specificity: 0.73	Hold-out	Stratified sampling	Total: 1778 diabetes: 279
[104]	Gupta et al. (2021)	Deep Neural Network	Precision: 0.90 accuracy: 0.95 sensitivity: 0.95 F1-score: 0.93 specificity: 0.95	Hold-out	None	Pima Indians
[105]	Roy et al. (2021)	Gradient boosting tree	Accuracy: 0.92 precision: 0.86 sensitivity: 0.87 specificity: 0.79 AUC (ROC): 0.84	Tenfold cross-validation	Correlation matrix SMOTE	Total: 500 diabetes: 289 age: 20–80 Imbalanced
[106]	Zhang et al. (2021)	Bagging boosting GBT, RF, GBM	Accuracy: 0.82 sensitivity: 0.85 specificity: 0.82 AUC (ROC): 0.89	Training set (80%) test set (20%) tenfold cross-validation	SMOTE	Total: 37,730 diabetes: 9.4% age: 50–70 Imbalanced
[107]	Turnea et al. (2018)	Decision tree	Accuracy: 0.74 sensitivity: 0.60 specificity: 0.82 RMSE: 26.1	Training set (75%) test set (25%)	None	Pima Indians
[108]	Vettoretti et al. (2021)	RFE-Borda	RMSE: 0.98	None	Correlation matrix	English longitudinal study of ageing

### Risk of bias analyses

For the risk of bias in the studies: unbalanced data means that the number of observations per class is not equally distributed. Some studies applied complementary techniques (e.g., SMOTE) to prevent the bias produced by unbalance in data. These techniques undersample the predominant class or oversample the minority class to produce a balanced dataset.

Other studies used different strategies to deal with other risks for bias. For instance, they might exclude specific age groups or cases presenting a second disease that could interfere with the model’s development to deal with the heterogeneity in some cohorts’ age.

For the risk of bias across the studies: the comparison between models was performed on those reporting the most frequently used validation metrics, i.e., accuracy and AUC (ROC). The accuracy is estimated to homogenize the criteria of comparison when other metrics from the confusion matrix were calculated, or the population’s knowledge is known. The confusion matrix is a two-by-two matrix containing four counts: true positives, true negatives, false positives, and false negatives. Different validation metrics such as precision, recall, accuracy, and F1-score are computed from this matrix.

Two kinds of complementary techniques were found. Firstly, techniques for balancing the data, including oversampling and undersampling methods. Secondly, feature selection techniques such as logistic regression, principal component analysis, and statistical testing. A comparison still can be performed between them with the bias caused by the improvement of the model.

## Discussion

This section discusses the findings for each of the research questions driving this review.

### RQ1: What kind of features makes up the database to create the model?

Our findings suggest no agreement on the specific features to create a predictive model for type 2 diabetes. The number of features also differs between studies: while some used a few features, others used more than 70 features. The number and choice of features largely depended on the machine learning technique and the model’s complexity.

However, our findings suggest that some data types produce better models, such as lifestyle, socioeconomic and diagnostic data. These data are available in most but not all Electronic Health Records. Also, retinal fundus images were used in many of the top models, as they are related to eye vessel damage derivated from diabetes. Unfortunately, this type of image is no available in primary care data.

### RQ2: What machine learning technique is optimal to create a predictive model for type 2 diabetes?

**Fig. 3:**
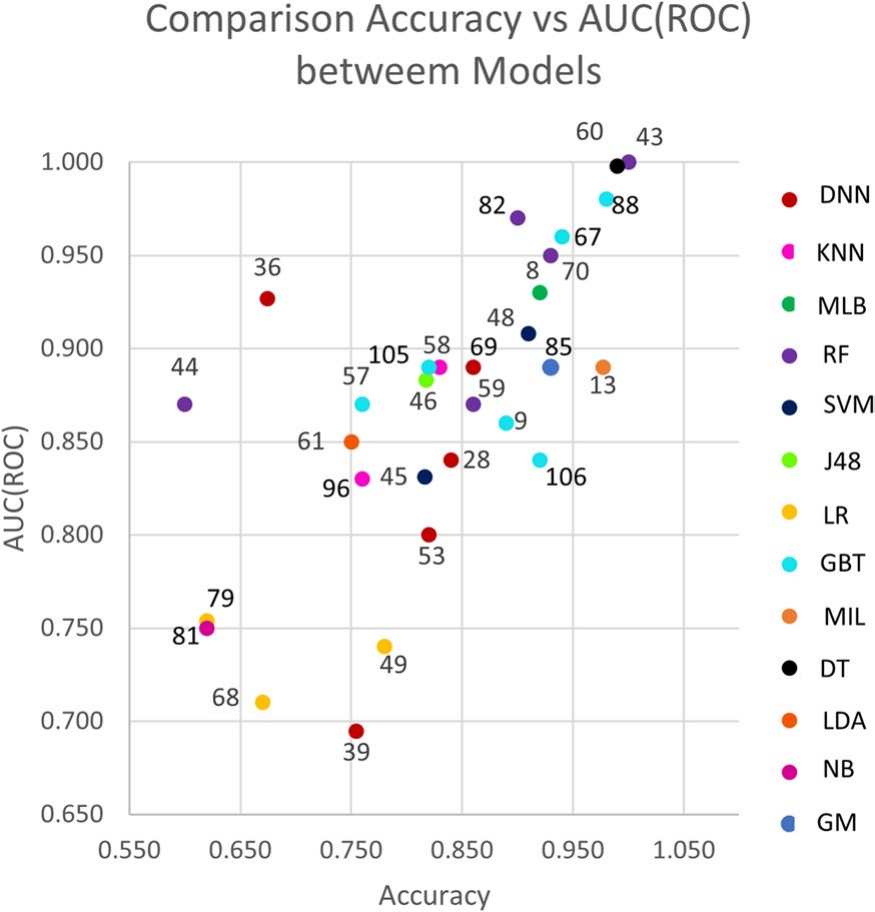
Scatterplot of AUC (ROC) vs. Accuracy for included studies. Numbers correspond to the number of reference and color dot the type of model, desired model has values of x-axis equal 1 and y-axis also equal 1

Figure 3 shows a scatter plot of studies that reported accuracy and AUC (ROC) values (x and y axes, respectively. The color of the dots represents thirteen of the eighteen types of model listed in the background. Dot labels represent the reference number of the study. A total of 30 studies is included in the plot. The studies closer to the top-right corner are the best ones, as they obtained high values for both validation metrics.

**Fig. 4:**
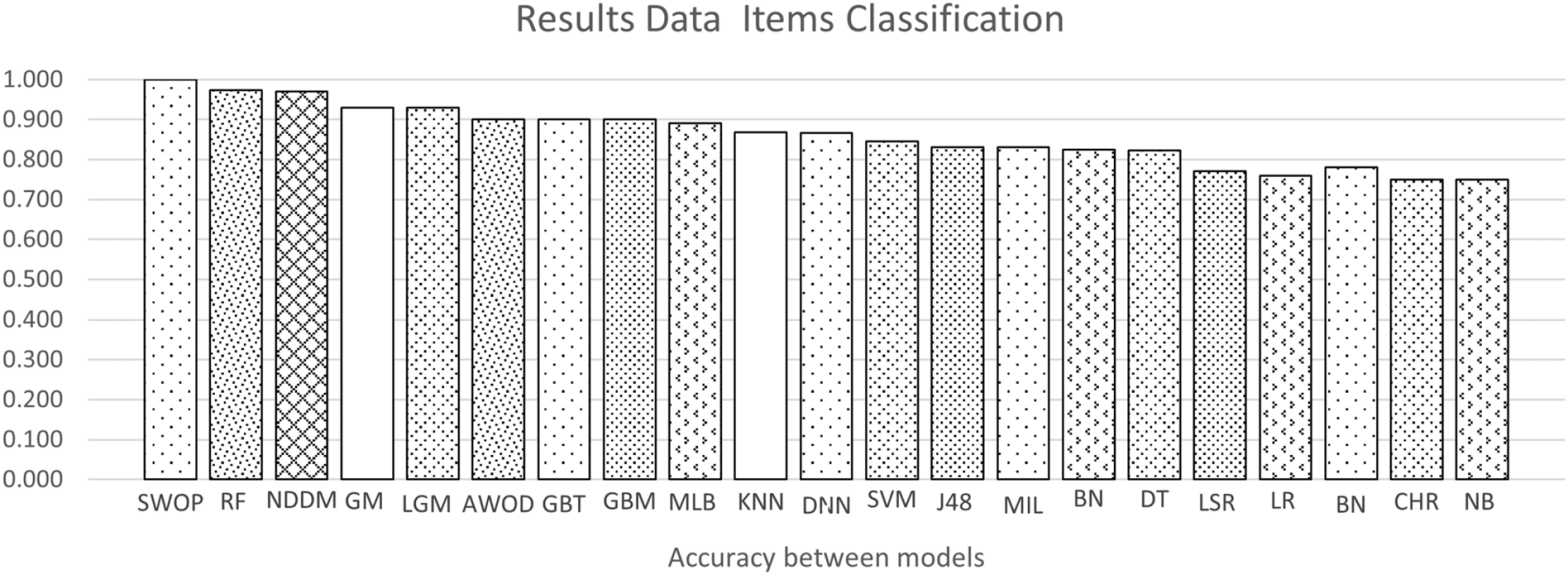
Average accuracy by model. For papers with more than one model the best score is the model selected to the graph. A better model has a higher value

**Fig. 5:**
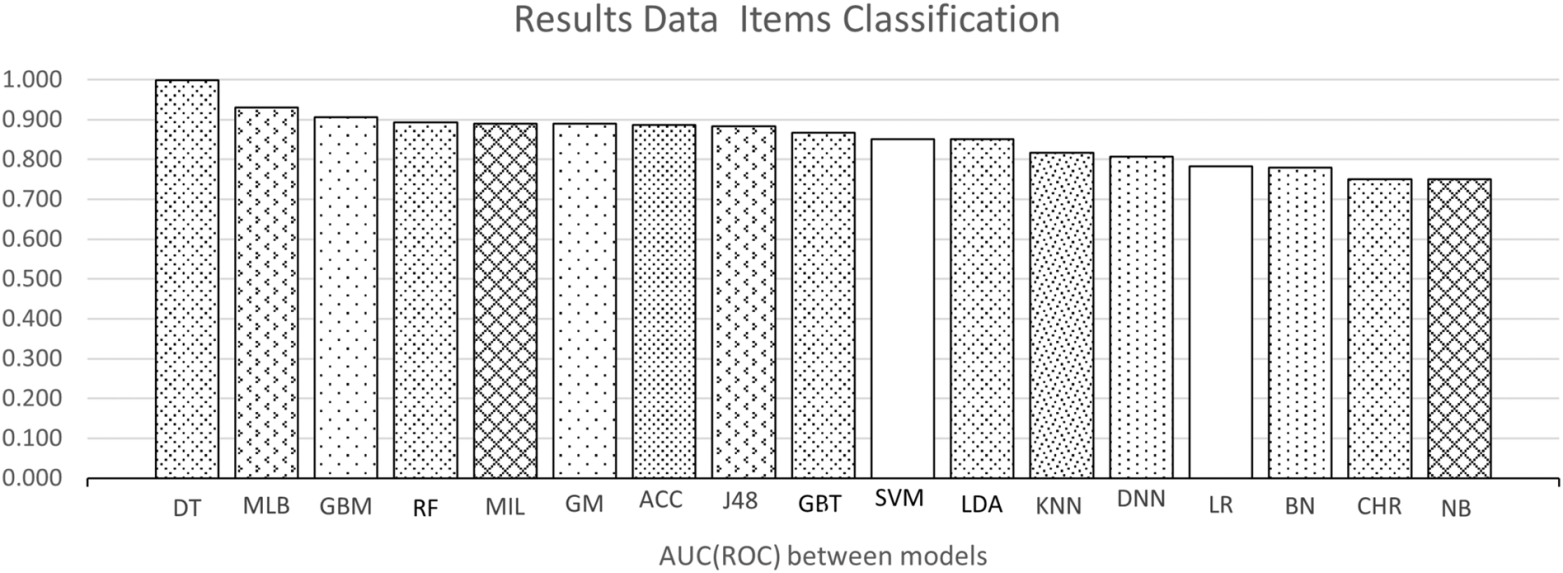
Average AUC (ROC) by model. For papers with more than one model the best score is the model selected to the graph. A better model has a higher value

Figures 4 and 5 show the average accuracy and AUC (ROC) by model. Not all models from the background appear in both graphs since not all studies reported both metrics. Notably, most values represent a single study or the average of two studies. The exception is the average values for SVMs, RFs, GBTs, and DNNs, calculated with the results reported by four studies or more. These were the most popular machine learning techniques in the included studies.

### RQ3: Which are the optimal validation metrics to compare the models’ improvement?

Considerable heterogeneity was found in this regard, making it harder to compare the performance between the models. Most studies reported some metrics computed from the confusion matrix. However, studies focused on statistical learning models reported hazard ratios and the c-statistic.

This heterogeneity remains an area of opportunity for further studies. To deal with it, we propose reporting at least three metrics from the confusion matrix (i.e., accuracy, sensitivity, and specificity), which would allow computing the rest. Additionally, the AUC (ROC) should be reported as it is a robust performance metric. Ideally, other metrics such as the F1-score, precision, or the MCC score should be reported. Reporting more metrics would enable benchmarking studies and models.

### Summary of the findings

- Concerning the datasets, this review could not identify an exact list of features given the heterogeneity mentioned above. However, there are some findings to report. First, the model’s performance is significantly affected by the dataset: the accuracy decreased significantly when the dataset became big and complex. Clean and well-structured datasets with a few numbers of samples and features make a better model. However, a low number of attributes may not reflect the real complexity of the multi-factorial diseases.
- The top-performing models were the decision tree and random forest, with an similar accuracy of 0.99 and equal AUC (ROC) of one. On average, the best models for the accuracy metric were Swarm Optimization and Random Forest with a value of one in both cases. For AUC (ROC) decision tree with an AUC (ROC) of 0.98, respectively.
- The most frequently-used methods were Deep Neural Networks, tree-type (Gradient Boosting and Random Forest), and support vector machines. Deep Neural Networks have the advantage of dealing well with big data, a solid reason to use them frequently [27, 28]. Studies using these models used datasets containing more than 70,000 observations. Also, these models deal well with dirty data.
- Some studies used complementary techniques to improve their model’s performance. First, resampling techniques were applied to otherwise unbalanced datasets. Second, feature selection techniques were used to identify the most relevant features for prediction. Among the latter, there is principal component analysis and logistic regression.
- The model that has a good performance but can be improved is the Deep Neural Network. As shown in Figure 4, their average accuracy is not top, yet some individual models achieved 0.9. Hence, they represent a technique worth further exploration in type 2 diabetes. They also have the advantage that can deal with large datasets. As shown in Table 2 many of the datasets used for DNN models were around 70,000 or more samples. Also, DNN models do not require complementary techniques for feature selection.
- Finally, model performance comparison was challenging due to the heterogeneity in the metrics reported.

## Conclusions

This systematic review analyzed 90 studies to find the main opportunity areas in diabetes prediction using machine learning techniques.

### Findings

The review finds that the structure of the dataset is relevant to the accuracy of the models, regardless of the selected features that are heterogeneous between studies. Concerning the models, the optimal performance is for tree-type models. However, even tough they have the best accuracy, they require complementary techniques to balance data and reduce dimensionality by selecting the optimal features. Therefore, K nearest neighborhoods, and Support vector machines are frequently preferred for prediction. On the other hand, Deep Neural Networks have the advantage of dealing well with big data. However, they must be applied to datasets with more than 70,000 observations. At least three metrics and the AUC (ROC) should be reported in the results to allow estimation of the others to reduce heterogeneity in the performance comparison. Therefore, the areas of opportunity are listed below.

### Areas of opportunity

First, a well-structured, balanced dataset containing different types of features like lifestyle, socioeconomically, and diagnostic data can be created to obtain a good model. Otherwise, complementary techniques can be helpful to clean and balance the data.

The machine learning model will depend on the characteristics of the dataset. When the dataset contains a few observations, machine learning techniques present a better performance; when observations are more than 70,000, Deep Learning has a good performance.

To reduce the heterogeneity in the validation parameters, the best way to do it is to calculate a minimum of three parameters from the confusion matrix and the AUC (ROC). Ideally, it should report five or more parameters (accuracy, sensitivity, specificity, precision, and F1-score) to become easier to compare. If one misses, it can be estimated from the other ones.

### Limitations of the study

The study’s limitations are observed in the heterogeneity between the models that difficult to compare them. This heterogeneity is present in many aspects; the main is the populations and the number of samples used in each model. Another significant limitation is when the model predicts diabetes complications, not diabetes.

## Data Availability

All data generated or analysed during this study are included in this published article and its references.

## Abbreviations

DNN: Deep Neural Network
RF: Random forest
SVM: Support Vector Machine
KNN: k-Nearest Neighbors
DT: Decision tree
GBT: Gradient Boosting Tree
GBM: Gradient Boost Machine
J48: J48 decision tree
LR: Logistic regression and stepwise regression
LDA: Linear and quadratric discriminant analysis
MIL: Multiple Instance Learning boosting
BN: Bayesian Network
LGM: Latent growth mixture
CHR: Cox Hazard Regression
LSR: Least-Square Regression
LASSO: Least absolute shrinkage and selection operator
SCAD: Smoothed clipped absolute deviation
MCP: Minimax concave penalized likelihood
ACC: Alternating Cluster and Classification
MLB: Machine learning-based method
SMOTE: Synthetic minority oversampling technique
AUC (ROC): Area under curve (receiver operating characteristic)
DR: Diabetic retinopathy
GM: Gaussian mixture
NB: Naive Bayes
AWOD: Average weighted objective distance
SWOP: Swarm Optimization
NDDM: Newton’s Divide Difference Method
RMSE: Root-mean-square error
